# Ophthalmology Training in Greece: Challenges, Realities, and Pathways Toward Modernization

**DOI:** 10.7759/cureus.99474

**Published:** 2025-12-17

**Authors:** Maria Sidiropoulou

**Affiliations:** 1 Department of Surgery, UNC Health, Chapel Hill, USA; 2 Department of Ophthalmology, General Hospital of Korinthos, Korinthos, GRC

**Keywords:** brain drain, competency-based curriculum, european board of ophthalmology, greece, ophthalmology training, residency education, simulation-based training, surgical education reform

## Abstract

This editorial examines the current state of ophthalmology training in Greece, situates it within the broader European landscape, and provides a narrative reflection on the challenges and potential reform pathways.

In the author's view, ophthalmology training in Greece remains rooted in a traditional apprenticeship model, with few structural reforms over the past decades. While much of Europe has transitioned toward competency-based training guided by the European Board of Ophthalmology (EBO) and the Union of European Medical Specialists (UEMS), Greece has struggled to keep pace. The persistence of waiting-list entry, time-based residency progression, limited surgical simulation, and lack of structured assessments may have created inequities in training and contributed to professional emigration. Greek training lags in adopting Entrustable Professional Activities (EPAs), programmatic assessment, and structured fellowships. Personal or individual reasons aside, the country's financial state and training-related inefficiencies have also driven the emigration of young ophthalmologists to other EU countries and beyond.

Reform is urgently required to align Greece with European standards. This should encompass the implementation of a national competency-based curriculum, digital surgical logbooks, mandatory simulation milestones, structured assessments, protected educational time, and international accreditation pathways. Beyond technical reforms, a cultural shift, recognizing education as equivalent to service provision, is essential to maintaining the ophthalmology workforce and enhancing patient outcomes.

## Editorial

Introduction

Ophthalmology has long been one of the most dynamic medical specialties, distinguished by its rapid adoption of technology, microsurgical precision, and constant evolution in therapeutics. Across Europe, training in ophthalmology has been reshaped by the efforts of the European Board of Ophthalmology (EBO) and the Union of European Medical Specialists (UEMS), culminating in harmonization initiatives such as the 2024 European Training Requirements [1-3]. Despite large variation between European countries, these reforms have emphasized competency-based frameworks, Entrustable Professional Activities (EPAs), and simulation-based skill acquisition, ensuring that graduates across the continent are better prepared for independent practice [3].

Yet, within this landscape of reform, Greece presents an instructive paradox. Despite its long tradition of medical excellence and its diaspora of highly regarded physicians abroad, Greece’s ophthalmology training system remains stubbornly traditional. Residents enter through a waiting-list mechanism that can delay training for years; curricula are poorly defined, simulation access is scarce, and structured assessments throughout residency are absent. A residency model prioritizing service provision over structured education might happen as a result.

This narrative explores the current state of ophthalmology training in Greece, comparing it to continental reforms and outlining the cultural, structural, and policy barriers that have hindered modernization. It also reflects on possible pathways forward, recognizing that aligning Greece with European and global standards is not only a technical necessity but also an ethical imperative.

Historical and structural context

The foundations of Greek residency training were laid in the mid-20th century, at a time when apprenticeship models dominated globally. Young doctors learned primarily by observation and opportunistic participation in surgery. While many European countries gradually introduced structured curricula and assessments in the late 20th century, Greece retained its reliance on hospital-based service provision as the central component of training.

The Ministry of Health regulates entry into ophthalmology training through a waiting-list system [4-6]. The waiting-list system for ophthalmology training in Greece is a distinctive feature of Greek postgraduate medical education [4,5]. After obtaining a medical degree and a license to practice, graduates who wish to specialize in ophthalmology must register on an official waiting list maintained by the Ministry of Health. The waiting period before starting ophthalmology training can be several years, depending on demand and the number of available training positions in accredited hospitals. This system is not unique to ophthalmology but applies to most medical specialties in Greece, and it has been associated with significant delays in career progression for young doctors. Entry into residency is not based on a competitive national examination or merit-based selection, but rather on the order of application - essentially a first-come, first-served system. Unlike competitive national examinations used in Spain, France, or the United Kingdom’s portfolio and interview system, Greece’s queue-based entry lacks merit-based allocation [7]. This not only delays the training of motivated young ophthalmologists but also undermines fairness and efficiency. The waiting-list approach has also been criticized for contributing to inefficiencies in workforce planning and for not considering academic performance or other qualifications. It also leads to heterogeneity in training experiences, as the quality and structure of residency programs can vary significantly between institutions [4,5,7]: rotations across subspecialties such as cornea, retina, glaucoma, and oculoplastics exist in larger hospitals, but smaller centers often lack full coverage, leaving residents dependent on variable exposure. With no nationally defined curriculum or milestones, the breadth and depth of training remain highly inconsistent. These issues have prompted calls for reform in Greek medical education, including the adoption of more transparent and merit-based selection processes.

Curriculum and competency development

The absence of a competency-based curriculum is the most significant structural weakness of Greek ophthalmology training. Progression is determined almost exclusively by time served and service fulfilled, not by demonstrated mastery of skills. Residents are rarely formally assessed, and, when feedback is offered, it is typically informal and dependent on the supervisor.

By contrast, the European Training Requirements emphasize EPAs, which define specific activities - such as performing uncomplicated phacoemulsification, managing acute glaucoma, or repairing a corneal laceration - that residents must be trusted to perform independently by graduation [3]. Certain European countries, inspired by the UK’s Ophthalmic Specialty Training curriculum, have embraced these frameworks and introduced programmatic assessments, e-portfolios, and workplace-based evaluations [8].

In Greece - and probably other countries with similar training models - the lack of these structures leaves residents uncertain about their progress and ill-equipped to benchmark themselves against international peers; an example highlighting this would be the lack of simulation or wet lab training requirements throughout residency.

Surgical exposure and training opportunities

Ophthalmology training relies heavily on the development of microsurgical skills, as nearly all major ophthalmic procedures - including cataract extraction, corneal transplantation, glaucoma surgery, vitreoretinal interventions, and ocular trauma repair - require precise manipulation under an operating microscope. Microsurgical skill acquisition is therefore becoming a core component of residency curricula, with structured training in wet labs, simulation models, and virtual reality platforms. These modalities have demonstrated efficacy in improving residents’ technical performance, confidence, and surgical accuracy, and are widely - albeit not universally - integrated into training programs [9-14].

Assessment of microsurgical proficiency is increasingly standardized, utilizing objective tools such as motion-tracking devices and video-based scoring systems, which correlate well with skill improvement after targeted training [10,11]. The importance of microsurgical skills is reflected in minimum surgical case requirements for board eligibility, and in the structure of competency-based curricula across Europe and internationally [15,16].

In Greece, however, microsurgical exposure is uneven. In high-volume university hospitals, residents may approach adequate numbers, but in many regional centers, opportunities are scarce. According to a recent EBO survey, more than 25% of European trainees self-reported performing no live cataract surgeries during residency, with the mean number of entire cataract procedures performed by residents in Greece among the lowest in Europe. This is consistent with broader European data, showing significant heterogeneity in surgical exposure, with many residents in Greece not meeting the minimum case numbers considered necessary for independent practice and self-confidence in cataract surgery [5,7,17].

This limited exposure is attributed to various factors. Lack of a standardized national curriculum, heterogeneity among training centers, and insufficient surgical case volume in many hospitals are some. Mainly, residents in major universities or general hospitals complete a realistically adequate number of cataract surgeries; those in smaller centers may have limited surgical experience [7]. Other systemic factors come into play, such as population distribution and resource availability. As a result, many Greek ophthalmology graduates seek additional fellowship training after residency to achieve surgical competence in cataract surgery [5,17,18].

Besides the above, the European Working Time Directive, which restricts working hours to 48 per week, has had variable implementation in Greece [19]. In practice, Greek surgery - including ophthalmology - residents often exceed these hours, yet paradoxically still lack protected surgical time, as service needs dominate, thus frequently ending up overworked but undertrained.

Simulation and the modernization gap

One disparity between Greece and some of its European counterparts lies in the use of simulation. Virtual reality cataract simulators (e.g., EyeSi) are now recognized as critical tools for training, offering safe, reproducible, and competency-based practice environments. Several European countries mandate simulation milestones before residents operate on live patients.

In Greece, simulation facilities are rare, confined to only a few academic centers, and not integrated into residency curricula. Wet labs exist sporadically but lack standardized curricula. The problem of relying on opportunistic learning would greatly diminish with frequent training opportunities on a simulator; this change would make a great difference in the residents' confidence levels in the operating room.

Assessment and professional development

Objective Structured Assessments of Technical Skills (OSATS) play a central role in evaluating microsurgical skills during ophthalmology residency training by providing a standardized, objective, and reproducible framework for assessment. OSATS and its ophthalmology-specific adaptations have demonstrated validity, reliability, and feasibility for both formative and summative evaluation of technical performance in microsurgical tasks, including cataract surgery and corneal suturing.

In Greece, residents are not required to pass in-training examinations, complete OSATS, or appear before competency committees. Progression is automatic with time, and the final licensing exam assesses theoretical knowledge more than clinical or surgical competency.

In contrast, in the rest of Europe, even though there is still significant heterogeneity, ophthalmology residency systems are increasingly sophisticated [7,17]. Programmatic assessment, combining multiple low-stakes evaluations such as mini-CEX (Mini Clinical Evaluation Exercise), DOPS (Direct Observation of Procedural Skills), simulation outcomes, and multi-source feedback, is used to make defensible high-stakes decisions. While voluntary for most EU ophthalmology trainees and not a standardized component of training, the EBO Diploma provides a supranational benchmark, recognized across Europe. Exams are taken by more than 700 candidates annually [19]. For Greek residents, the EBOD can serve as a valuable external validation, but it remains optional and self-driven.

Research and academic development

In many European countries, university-affiliated residency programs routinely include research activities, academic teaching, and opportunities for scholarly output as part of the formal curriculum. Residents are often expected to participate in clinical or laboratory research, present at conferences, and publish in peer-reviewed journals, with research experience sometimes considered in selection criteria for training positions [20,21]. Post-residency fellowships in Europe also frequently involve substantial research components, and institutional support for academic engagement is increasing, especially in university hospitals [21].

Contrary to frameworks in some other EU countries, where scholarly activity is increasingly embedded in training curricula, ophthalmology residents in Greece face limited encouragement to engage in research or quality improvement. The lack of structured academic engagement further compromises Greece’s visibility in international ophthalmology research.

Brain drain phenomenon

Perhaps the most profound consequence of these structural deficiencies is the sustained emigration of Greek ophthalmologists, also known as brain drain [22-24]. This migration is dually driven by financial reasons - persistent unemployment, job insecurity, reduced salaries, over-taxation, and limited research funding within the Greek healthcare system - as well as structural issues in Greek ophthalmology training - long waiting lists for residency entry, variable surgical exposure, and lack of standardized academic requirements. EU mobility frameworks, such as Directive 2005/36/EC, facilitate recognition of qualifications, making it easier for Greek ophthalmologists to relocate to Germany, the UK, or Scandinavia [25].

While emigration provides individuals with opportunities, it depletes the domestic healthcare system. Hospitals struggle to retain talent, and patients face longer waiting times and variability in care. The cycle perpetuates itself: as talented residents leave, domestic programs lose role models and mentors, further weakening the system.

Future directions: a roadmap for reform

Reforming ophthalmology training in Greece requires both structural and cultural shifts. Key steps include: (1) National Competency-Based Curriculum: Adoption of EPAs aligned with the 2024 ETR, with clearly defined milestones and entrustment criteria. (2) Digital Surgical Logbook: A centralized, faculty-verified logbook to track case numbers and ensure minimum cataract and subspecialty exposure. (3) Simulation Integration: Establishment of simulation hubs across teaching hospitals, mandating simulator proficiency before live cataract surgery. (4) Structured Assessments: Implementation of OSATS, mini-CEX, and DOPS, with Clinical Competency Committees providing biannual resident reviews. (5) Protected Educational Time: Weekly sessions for structured teaching, journal clubs, morbidity and mortality meetings, and simulation practice. (6) Research Integration: Mandating at least one scholarly project during residency, supported by mentorship and resources. (7) International Accreditation: Pursuing alignment with Accreditation Council for Graduate Medical Education (ACGME)-International or EBO accreditation, raising global recognition of Greek programs. (8) Exchange Programs: Funded short-term fellowships and bilateral agreements to expose residents to high-volume European centers while attracting fellows to Greece. (9) Faculty Development: Training supervisors in competency-based education, assessment, and simulation debriefing, while recognizing teaching in promotion criteria.

Conclusion

Ophthalmology training in Greece sits at a crossroads. While some EU countries are advancing toward competency-based, structured, and simulation-driven training, Greece retains a traditional service-based model. The consequences - variability in surgical competence, lack of structured assessments, limited research engagement, and widespread emigration - are stark.

Reform is both urgent and feasible. By adopting a competency-based curriculum, mandating simulation, standardizing assessments, and investing in research and international partnerships, Greece can revitalize its ophthalmology training. Most importantly, cultural change must accompany structural reform, ensuring that residents are valued as learners and future leaders, not just service providers. Modernization is not optional. It is an ethical obligation to patients, a professional duty to residents, and a national necessity to retain talent and ensure Greece’s contribution to global ophthalmology. The time for reform is now.
